# Patient-Reported Outcome Coordinator Did Not Improve Quality of Life Assessment Response Rates: A Report from the Children's Oncology Group

**DOI:** 10.1371/journal.pone.0125290

**Published:** 2015-04-27

**Authors:** Donna Johnston, Robert Gerbing, Todd Alonzo, Richard Aplenc, Rajaram Nagarajan, Fiona Schulte, Patricia Cullen, Lillian Sung

**Affiliations:** 1 Division of Hematology/Oncology, Children’s Hospital of Eastern Ontario, Ottawa, Ontario, Canada; 2 Children’s Oncology Group, Monrovia, California, United States of America; 3 Department of Preventive Medicine, University of Southern California, Los Angeles, California, United States of America; 4 Pediatric Oncology/Stem Cell Transplant, Children's Hospital of Philadelphia, Philadelphia, Pennsylvania, United States of America; 5 Division of Oncology, Cincinnati Children’s Hospital Medical Center, Cincinnati, Ohio, United States of America; 6 Departments of Oncology and Pediatrics, University of Calgary, Calgary, Alberta, Canada; 7 Loretto Heights School of Nursing, Regis University, Denver, Colorado, United States of America; 8 Division of Haematology/Oncology, The Hospital for Sick Children, Toronto, Ontario, Canada; Emory University/Georgia Insititute of Technology, UNITED STATES

## Abstract

**Purpose:**

Health related quality of life (HRQL) assessments during therapy for pediatric cancer provide valuable information to better understand the patient experience. Our objective was to determine the impact of a patient-reported outcome (PRO) coordinator on HRQL questionnaire completion rates during a pediatric acute myeloid leukemia (AML) trial.

**Methods:**

AAML1031 is a multicenter Children’s Oncology Group therapeutic trial for *de novo* AML with a secondary aim to assess HRQL of children and adolescents treated with chemotherapy and hematopoietic stem cell transplantation (HSCT). Parents/guardians are the primary respondents and four questionnaires are administered at eight time points. The questionnaires are the PedsQL 4.0 Generic Core Scales, PedsQL 3.0 Acute Cancer Module, PedsQL Multidimensional Fatigue Scale, and the Pediatric Inventory for Parents. To improve response rates, a central PRO coordinator was instituted and reminded sites about upcoming and delinquent questionnaires. The proportion of HRQL questionnaires completed were compared prior to, and following institution of the PRO coordinator. This analysis evaluated the first five assessment time points.

**Results:**

There were231 families who consented to participate in the HRQL aim. Overall response rates for all questionnaires were 73–83%. At time point 1, within 14 days of chemotherapy initiation, post-PRO coordinator completion rates were significantly higher for three of four questionnaires. However, the effect was not sustained and at time point 4, one month following last chemotherapy or HSCT, completion rates were significantly lower post-PRO coordinator for all four questionnaires.

**Conclusion:**

Addition of a central PRO coordinator did not result in sustained improvement in HRQL questionnaire completion rates. Efforts to improve response rates must consider other strategies.

## Introduction

Health related quality of life (HRQL) assessments during therapy for pediatric cancer provide valuable information to better understand the patient experience. They also provide information that allows clinicians to identify if, when, and how interventions intended to improve HRQL should be instituted [1,2]. When HRQL is measured on different treatments regimens, it provides insight into how the regimens differ from the patient’s and family’s perspective. This information can subsequently be used to help clinicians and families when choosing a treatment strategy [2–4].

AAML1031 (NCT 01371981) was a Phase 3 Children’s Oncology Group (COG) multi-center trial for *de novo* AML that randomized patients to receive or not receive bortezomib. Patients were risk stratified and low risk patients received four courses of chemotherapy, namely Induction I, Induction II, Intensification I, and Intensification II. Patients with high risk disease received the first three courses of chemotherapy followed by best allogeneic donor hematopoietic stem cell transplantation (HSCT). Thus, all patients on AAML1031 received three courses of chemotherapy and then either Intensification II chemotherapy or HSCT. This study was approved by the Institutional Review Board at all participating institutions and all parents provided written informed consent to participate in the study and for their children’s data to be used for research purposes (see S1 Table for a list of participating institutions).

One of the embedded secondary aims of AAML1031 assessed the HRQL of children and adolescents treated with chemotherapy and HSCT, and also described parental stress in this population. We have recently described reasons for non-response in the context of this trial and the major themes identified were: patient too ill; passive or active refusal by respondent; developmental delay; logistical challenges; and poor knowledge of study processes from both the respondent and institutional perspective [5].

One of the common challenges in conducting studies of HRQL, particularly for multi-center studies, is ensuring high response rates. Non-response is problematic because of the potential for bias and loss of power. Recent studies of HRQL in pediatric oncology have had response rates as low as 58% [6–11]. Several studies have demonstrated that having a coordinating center that provides feedback to participating centers aids greatly in the completeness and quality of data provided [12, 13]. As well, a recent COG study examining the role of the Clinical Research Associate (CRA) in cancer control studies highlighted the need for staff to support these studies [14].

For the HRQL aim of AAML1031, a patient-reported outcome (PRO) coordinator was instituted part-way through the study. One of the goals of the PRO coordinator was to maximize response rates for HRQL assessments on this study. While the incorporation of a central coordinator seemed to be a reasonable approach, to our knowledge, this approach has never been rigorously evaluated to determine whether it does improve response rates. Therefore, the objective of this study was to determine whether the institution of a PRO coordinator improved HRQL questionnaire completion rates during a multi-center co-operative group clinical trial.

## Methods

Three instruments were used for HRQL assessment: the PedsQL 4.0 Generic Core Scales [15, 16], PedsQL 3.0 Acute Cancer Module [15], and PedsQL Multidimensional Fatigue Scale [17, 18]. The PedsQL 4.0 Generic Core Scales is a multidimensional instrument that measures physical functioning, emotional functioning, social functioning and school functioning dimensions. The PedsQL 3.0 Acute Cancer Module measures pain and hurt, nausea, procedural anxiety, treatment anxiety, worry, cognitive problems, perceived physical appearance, and communication. The PedsQL Multidimensional Fatigue Scale measures general fatigue, sleep/rest fatigue and cognitive fatigue. All these scales are available in a proxy-respondent (ages 2–18 years) and self-response (ages 5 to 18 years) format. Parental stress was measured using the Pediatric Inventory for Parents which measures parenting stress related to caring for a child with an illness [19,20]. All instruments are reliable and valid in pediatric cancer patients [15–20]. These 4 questionnaires were administered at each assessment time point. The estimated time for completion of all 4 questionnaires was 23–32 minutes. The questionnaires could be completed in any order and partial submissions were permitted (for example, submission of only 3 or less questionnaires). The protocol stipulated eight assessment time points as follows: (1) within 14 days of Induction I initiation; (2) ≥ day 21 of Induction II, but prior to start of Intensification I; (3) ≥ day 21 of Intensification I, but prior to start of Intensification II; (4) 1 month (± 7 days) from start of Intensification II or HSCT; (5) 4 months (± 1 month) from start of Intensification II or HSCT; (6) 12 months (± 1 month) from date of diagnosis; (7) 24 months (± 3 months) from date of diagnosis; and (8) 36 months (± 3 months) from date of diagnosis. The time points for the HRQL assessments correlated with times when the patient would either be hospitalized or due for a clinic visit in order to aid in compliance with completing the questionnaires.

The parent or guardian provided proxy assessments for all patients, while child self-report was optional. Eligibility for the HRQL aim included between 2 and 18 years of age and a parent or guardian who could read English. For self-report scores, children had to be ≥ 5 years of age and be able to understand English. Age-appropriate questionnaires were downloaded from the COG website by institutional CRAs for all consenting participants and the completed questionnaires were reviewed by the CRA for completeness. There was at least one CRA at each institution and the median number of CRAs at each site in COG is 5 (range 1 to 34). CRAs were responsible for reminding families to complete the questionnaires within the specified time range, and followed up with families with delinquent questionnaires. All responses were entered into the COG database via remote data entry with the original questionnaires retained by the institutions. If respondents did not complete a questionnaire (parent or child) at a particular time point, they still continued to participate in subsequent time points. The respondents in the study continued to submit HRQL assessments until one of the following: they completed all time point questionnaires; consent to participate in the HRQL study was withdrawn; or the patient was removed from AAML1031 protocol therapy (for reasons such as death, relapse, or refractory disease).

### Effect of a Patient-Reported Outcome Coordinator

In order to improve response rates to the HRQL aim, a central PRO study coordinator was instituted. The PRO coordinator contacted sites with enrolled patients to remind them of upcoming HRQL assessments as well as to follow-up in cases of delinquency of HRQL data. The PRO coordinator evaluated for delinquent data once a month. In the case of delinquent data, the PRO coordinator sent an email upon discovery of the delinquency to encourage submission of the data. In sites where there continued to be delinquency within the next month, then the PRO coordinator sent a second email to the site. In addition, in the case of incomplete HRQL questionnaires, the PRO coordinator contacted the site and asked them to address the deficiency. All contact by the PRO coordinator was made with the CRA at the institution, and the PRO coordinator did not contact families directly.

The proportion of HRQL questionnaires completed prior to the hiring of the PRO coordinator was compared to the number completed after the coordinator began contacting sites to remind them of upcoming completion deadlines. Only parental questionnaire completion rates were used in this analysis since child self-report was optional.

### Statistics

AAML1031 opened to accrual on June 20, 2011 and was still enrolling patients as of March 31, 2013, the cut-off date for this analysis. The central PRO coordinator began contacting sites on August 9, 2012. For all patients participating in the HRQL aim, each response time point was classified as: (1) Pre-PRO coordinator, if the latest allowable date for submission of HRQL data was before August 9, 2012; (2) Post-PRO coordinator, if the earliest possible date for submission was after August 9, 2012; and (3) Indeterminate, if the range of allowable submission dates included August 9, 2012. The analysis excluded the indeterminate patients. Each HRQL questionnaire was categorized as completed or not. Questionnaires that were not completed but were within 30 days past the maximum completion time as of March 31, 2013 were not yet considered delinquent and were excluded from the analysis. Only time points 1–5 were included in this analysis because too few patients had reached time point 6 (n = 4). The Pearson chi-squared test was used to compare differences in proportions of questionnaires; Fisher’s exact test was used when data were sparse. All tests were two sided and statistical significance was set at alpha = 0.05. Analysis was conducted using the SAS statistical program (SAS-PC, version 9.2; SAS Institute Inc).

## Results

As of the cut-off date of March 31, 2013, there were 402 eligible patients enrolled to AAML1031. Of these patients, 235 consented to participate in the HRQL aim. Four patients were excluded because they did not read English, thus 231 participated. There were 67 enrolled patients who were removed from study and did not complete all of the HRQL assessments through time point 5 due to either withdrawing from the study before completing protocol treatment (n = 51) or withdrawing consent to further participate in the HRQL aim (n = 16). In terms of classifying all proxy-reported time points for the PedsQL 4.0 Generic Core Scales, there were 298 classified as pre-PRO coordinator, 632 cases classified as post-PRO coordinator (may not be delinquent depending on maximum completion date), and 42 classified as indeterminate. For assessment time point 1 and the PedsQL 4.0 Generic Core Scales, 118 were pre-PRO coordinator, 93 were post-PRO coordinator, 8 were indeterminate and 12 were not yet delinquent. The other 3 modules had similar numbers as the PedsQL 4.0 Generic Core Scales.

Table 1 shows the proportion of questionnaires that were completed pre- and post-PRO coordinator institution. The overall response rates ranged from 73–83% and in general the highest response rate were seen at time point 1 and declined over time. At time point 1 (within 14 days of Induction I), the PedsQL 4.0 Generic Core Scale, PedsQL Cancer Module and Peds QL Multidimensional Fatigue Scale had significantly higher completion rates post PRO-coordinator compared to pre PRO-coordinator. However, the difference was not sustained and at time point 4 (one month from start of Intensification II or HSCT), all four questionnaires had significantly lower completion rates post-PRO coordinator implementation. All assessments had similar completion rates at the specific time points.

**Table 1:** Proportion of assessments completed and not completed pre and post PRO coordinator institution.

		Pre-PRO Coordinator			Post-PRO Coordinator			Overall	
Instrument	Time Point	Completed	Not Completed	Completion Rate (%)	Completed	Not Completed	Completion Rate (%)	Completion Rate (%)	Pre-PRO vs. Post-PRO P Value
PedsQL 4.0 Generic Core Scale	1	92	26	78	84	9	90	83	**0.017**
	2	63	15	81	70	16	81	81	0.919
	3	44	9	83	57	13	81	82	0.820
	4	32	4	89	45	24	65	73	**0.009**
	5	9	4	69	36	11	77	75	0.719
PedsQL 3.0 Cancer Module	1	90	28	76	83	10	89	82	**0.015**
	2	62	16	79	70	16	81	80	0.758
	3	45	8	85	57	14	80	82	0.505
	4	32	4	89	45	25	64	73	**0.007**
	5	9	4	69	36	11	77	75	0.719
PedsQL Multidimensional Fatigue Scale	1	88	30	75	83	10	89	81	**0.007**
	2	62	16	79	70	16	81	80	0.758
	3	43	10	81	57	14	80	81	0.906
	4	32	4	89	45	25	64	73	**0.007**
	5	9	4	69	36	11	77	75	0.719
Pediatric Inventory for Parents	1	93	25	79	82	12	87	83	0.109
	2	61	17	78	67	19	78	78	0.963
	3	44	9	83	57	14	80	81	0.698
	4	32	4	89	45	25	64	73	**0.007**
	5	9	4	69	36	12	75	74	0.728

## Discussion

We found that institution of a central PRO-coordinator led to improvement in HRQL questionnaire response rates only with the first assessment time point, within 14 days of Induction I initiation. However, contacting sites did not lead to a sustained improvement in HRQL questionnaire completion rates and in fact, post-PRO coordinator, there was a significantly lower completion rate one month from start of intensification II or HSCT. This observation is contrary to previously published studies which recommended or demonstrated that a coordinating center improved the quality and completeness of data [12–14].

There are several potential explanations for our findings of a lack of sustained improvement in completion rates with the institution of a PRO-coordinator. First, the main benefit of a PRO coordinator is presumed to be related to reminding the site about the assessment time point. It is possible that sites do not have trouble remembering to obtain HRQL assessments at the assigned time and they may have tools available to ensure that time points are not missed. This hypothesis is supported by our previous study in which we found that patient and family factors such as acuity of illness and refusal of the respondent are the most important factors influencing response rates [5]. Second, there may be confounding by time. It is possible that response rates may decline over time if centers are more enthusiastic about the study at trial initiation. However, the converse may also be true and response rates may improve over time as centers become more familiar with a study. We are not aware of data which support either hypothesis. It is notable that the completion rate at time point 5 was the lowest of all the time points both pre and post PRO coordinator, and thus confounding by time may be the explanation for the decline post PRO coordinator at time point 4 noted in this study. Third, it is possible that a central PRO coordinator may be able to improve questionnaire response rates using other approaches and that our approach was sub-optimal.

There are limitations to this study that must be taken into consideration when interpreting our data. First, when the PRO coordinator began in the position, she contacted sites that had delinquent HRQL questionnaires at that time. It is possible that the PRO coordinator could have influenced these pre-PRO coordinator assessments. However, since questionnaires must be completed with pre-specified, protocol-dictated time frames, the only way the PRO coordinator could have influenced response rates for assessments due pre-PRO coordinator is if the assessments were actually completed on time but the data were not submitted to COG until after the PRO reminder. Second, the demographic information on those who completed the questionnaires pre and post-PRO coordinator are not available as the study is ongoing, and this could potentially aid in comparing the two groups. Finally, we do not have insight into the effect of the PRO coordinator on very late time points, which are typically the most difficult to obtain.

The strength of this study is the ability to evaluate the effect of a central PRO coordinator on response rates in a large, multi-site and multi-national trial. The diversity of sites and geographical locations increases the generalizability of our findings.

Future work should consider other potential strategies to improve response rates. One strategy is to implement electronic patient reported outcome (ePRO) systems. Electronic PRO systems utilize electronic data capture methods to assess issues that patients are able to report about themselves [21]. Some potential benefits of ePROs are less administrative burden, higher patient acceptance, easier implementation of skip patterns, the avoidance of secondary data entry errors, more accurate and complete data, and electronic scoring [22]. Another potential strategy to improve response rates would be to have a central coordinator contact families directly, rather than indirectly communicating with families through the site CRAs. In this study, the PRO coordinator did not contact families directly because of privacy considerations. However, the use of ePROs would allow direct communication with families who permitted email contact.

In conclusion, we were unable to demonstrate that a central PRO coordinator resulted in a sustained improvement in HRQL questionnaire completion rates on a pediatric AML therapeutic study. More study in this area is needed to confirm this finding. Other strategies, such as ePROs may be a potential mechanism to improve completion rates for pediatric oncology HRQL studies.

## Supporting Information

S1 Table List of Centers with AAML1031 REB Approval. (DOC)
